# “One Health” or Three? Publication Silos Among the One Health Disciplines

**DOI:** 10.1371/journal.pbio.1002448

**Published:** 2016-04-21

**Authors:** Kezia R. Manlove, Josephine G. Walker, Meggan E. Craft, Kathryn P. Huyvaert, Maxwell B. Joseph, Ryan S. Miller, Pauline Nol, Kelly A. Patyk, Daniel O’Brien, Daniel P. Walsh, Paul C. Cross

**Affiliations:** 1 Department of Biology, Pennsylvania State University, University Park, Pennsylvania, United States of America; 2 School of Biological Sciences, University of Bristol, Bristol, United Kingdom; 3 Department of Veterinary Population Medicine, University of Minnesota, St. Paul, Minnesota, United States of America; 4 Department of Fish, Wildlife, and Conservation Biology, Colorado State University, Fort Collins, Colorado, United States of America; 5 University of Colorado Boulder, Department of Ecology and Evolutionary Biology, Boulder, Colorado, United States of America; 6 United States Department of Agriculture, Animal and Plant Health Inspection Service, Veterinary Services, Science Technology and Analysis Services, Fort Collins, Colorado, United States of America; 7 United States Department of Agriculture Animal and Plant Health Inspection Service, Veterinary Services, National Wildlife Research Center, Fort Collins, Colorado, United States of America; 8 Wildlife Disease Laboratory, Michigan Department of Natural Resources, Lansing, Michigan, United States of America; 9 U.S. Geological Survey, National Wildlife Health Center, Madison, Wisconsin, United States of America; 10 U.S. Geological Survey, Northern Rocky Mountain Science Center, Bozeman, Montana, United States of America; Imperial College London, UNITED KINGDOM

## Abstract

The One Health initiative is a global effort fostering interdisciplinary collaborations to address challenges in human, animal, and environmental health. While One Health has received considerable press, its benefits remain unclear because its effects have not been quantitatively described. We systematically surveyed the published literature and used social network analysis to measure interdisciplinarity in One Health studies constructing dynamic pathogen transmission models. The number of publications fulfilling our search criteria increased by 14.6% per year, which is faster than growth rates for life sciences as a whole and for most biology subdisciplines. Surveyed publications clustered into three communities: one used by ecologists, one used by veterinarians, and a third diverse-authorship community used by population biologists, mathematicians, epidemiologists, and experts in human health. Overlap between these communities increased through time in terms of author number, diversity of co-author affiliations, and diversity of citations. However, communities continue to differ in the systems studied, questions asked, and methods employed. While the infectious disease research community has made significant progress toward integrating its participating disciplines, some segregation—especially along the veterinary/ecological research interface—remains.

## Abstract 2

"One Health" is a global initiative fostering interdisciplinary collaborations to address health challenges. Although an empirical analysis of published disease models shows a rapid growth in cross-disciplinary "One Health" collaborations, the continued segregation of medical, veterinary, and ecological communities poses an ongoing challenge.

## Author Summary

A cohesive reaction to emerging threats requires the efficient dissemination of knowledge and methodologies across multiple disciplinary boundaries. A “One Health” approach has been advocated to facilitate cross-disciplinary communication and research in responding to challenges in human, animal, and environmental health. We empirically describe collaboration networks surrounding one aspect of this One Health initiative: infectious disease transmission models. A systematic literature review and social network analysis showed that dynamic disease modelling grew faster than most biological and natural science disciplines over the last 25 y, yet the field remains somewhat compartmentalized. Many researchers contribute to a broad, cross-disciplinary group of journals, but publications from veterinarians and ecologists remain segregated into relatively discrete communities. While the veterinary and ecological communities regularly reference the broad, cross-disciplinary group, they only rarely cite each other; furthermore, the highest-tiered medical journals contributed very few papers to our search. Therefore, our results are somewhat mixed: while One Health may have precipitated a great deal of research interest in infectious disease, it has not fully overcome the barriers that segregate its contributing disciplines.

## Introduction

Understanding and containing emerging infectious diseases (EIDs), especially those crossing the wildlife, human, and domestic animal interfaces, is a global health imperative. Novel diseases have major implications on geopolitics [1–3], animal and human well-being, and wildlife conservation [4–6], and effective management hinges on understanding disease emergence and persistence processes. Disentangling the mechanisms underlying these processes is a fundamentally transdisciplinary endeavor, and disease research efforts must blend expertise on the behavior, health, and immune dynamics of a variety of host–pathogen pairs [7,8].

Efficiently disseminating knowledge and methodologies across disciplinary boundaries is essential for a cohesive reaction to emerging threats. However, researchers tend to organize themselves into discipline-specific “silos” that contain robust internal research communities, but that only rarely interact with one another. This is particularly true of the disciplines studying infectious disease: workplaces range from hospitals, to microbiological laboratories, to ecological field sites, to mathematical computing facilities, and communicating across these physical and cultural boundaries is difficult [9]. Modern ecological, epidemiological, medical, and veterinary programs advocate “One Health” approaches to facilitate cross-disciplinary communication and research [10]. One Health targets three basic objectives: (1) achieving human health, (2) achieving animal health, and (3) developing resilient, sustainable ecosystems [11]. Although research disciplines vary in the relative importance they ascribe to each objective, all objectives should retain value within each discipline.

Despite efforts to integrate, we continue to observe limited participation by all relevant viewpoints in our collective work on diseases breaching the domestic livestock–wildlife interface, leading us to question One Health’s efficacy. While recent papers discuss One Health applications and perspectives [8,12], we are not aware of empirical analyses measuring temporal collaboration trends within the infectious disease research community. Here, we fill that gap with a quantitative study of cross-disciplinary research patterns surrounding one important aspect of One Health: between-host zoonotic pathogen transmission.

In order to control our database's size, we confined our study to dynamic disease transmission models. Dynamic transmission models are integral to understanding and managing zoonotic disease [13,14]. While dynamic modeling is not the sole objective of One Health, model development requires integrating empirical and theoretical knowledge about pathogen virulence, ecological context (population densities, interaction patterns, and so on), environmental setting, and animal immunology and physiology. Experts at each level of the system contribute meaningful information that is distinct from knowledge at other levels. Model building requires sustained communication and cooperation across disciplinary boundaries, so we assumed that collaborative patterns surrounding dynamic models form a good proxy for collaborative patterns across the broader One Health spectrum.

We hypothesized that growth of the One Health initiative was associated with an erosion of traditional academic disciplinary “silos.” In particular, we expected the number of papers fulfilling our search to grow at a rate faster than overall growth rates in the biological and medical sciences. We suspected citations in recently published papers would reference a wider spectrum of disciplines than similar work published earlier. We also anticipated that journals today publish papers led by authors from more academic domains, and that individual authors now publish in more domains than in the past. Finally, we posited that approaches, methodologies, and research questions are increasingly shared across all contributing silos, indicating improved communication across historic disciplinary boundaries.

## Results

### Journals Divided into Two Traditional Disciplines and a Diverse Third Group

A literature search using Web of Science returned 2,258 papers containing dynamic disease transmission models; examination of titles and abstracts reduced this number to 1,628 suitable papers published in 108 journals, with 4,219 unique authors. We described citation patterns between journals using a network with nodes representing journals and directed, weighted edges representing citation rates between journals. Descriptive metrics associated with the journal network, as well as metrics from networks describing relationships between authors and between publications, are shown in S5 Table. We used a walk-trap algorithm [15], which accounted for both undirected and weighted edges, to identify communities in the journal network. We compared clustering under the walk-trap algorithm to clustering under a variety of other algorithms for undirected networks (fast-greedy and spin-glass communities, Louvain methods, and so forth).

**Fig 1:**
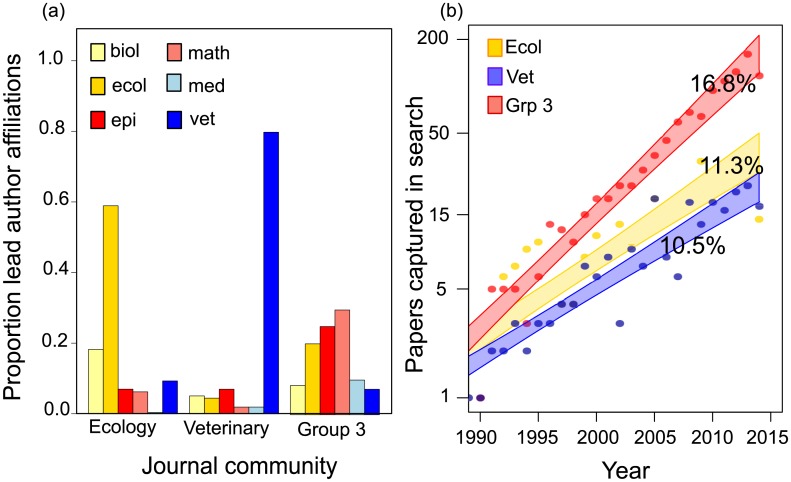
Participant diversity and publication growth. (A) Proportion of lead author affiliation disciplines across all 1,551 papers published in journals in the three major journal communities. “Math” here encompasses “math” and “stat” affiliations; “ecol” encompasses “eco,” “evo,” and “biol” affiliations; “vet” captures “vet,” “animal health,” and “animal science;” “Med” captures “med” and pharmacy affiliations. (B) Number of papers captured by our search through time. Blue = veterinary community; gold = ecology community; red = group 3. Numbers are the annual percent growth rate within each community. Data to generate this figure are contained in S1 Data.

The walk-trap algorithm suggested that journals segregated into three communities (S1 and S2 Figs), two of which corresponded to easily identifiable subject areas: ecology and veterinary medicine. Participants in a third community, which contained the preponderance of our papers, represented a wide range of disciplines (Fig 1A). Since this group defied a clear disciplinary label, we refer to it as “Group 3” throughout. Most algorithms that relied on undirected edges (which is to say, a reference from journal A to journal B was equivalent to a reference from journal B to journal A) partitioned Group 3 into separate epidemiological and mathematical biology communities (S3 Data contains a dataset of journal classifications under various methods). However, the walk-trap algorithm is a better approach for our purposes, since we are interested in identifying journal groups with reciprocal, rather than directional, citations. A full list of journals in the three largest communities is included on S1 Text, page 7.

The three largest communities encompassed 79 journals capturing 95.3% (1,551 of 1,628) of the papers returned by our search. Veterinarians tended to lead papers in the veterinary journal community; ecologists and evolutionary biologists tended to lead papers in the ecology community; and mathematicians, statisticians, or health-informatics experts disproportionately led papers in Group 3 journals (Fig 1A). In the veterinary community and Group 3, the majority of citations were directed toward papers within the same community, with 30.2% and 15.5%, respectively, of citations pointing outside the community. In the ecology community, the division between within-community and outside-community citations was close to equal, with 51.7% of citations pointing outside the community (S6 Table). A list of the ten most-cited papers in each community is provided in the online supplement (S1 Text, page 10).

### Number of Publications and Author Diversity Both Increased with Time

The number of papers fulfilling our search increased at a rate of 14.6% per year (95% CI [13.0%, 16.2%], Fig 1B, S7 Table) and doubled approximately every 5.06 y (95% CI [4.62, 5.66]). This puts publication growth rates for our search well above publication growth rates for most biological subdisciplines (e.g., a 10.4% growth rate for ecology and evolution [16]) and for medicine and health sciences (growth rate estimated around 8%–9% [17]). Lead author diversity—low when all lead authors in a community have the same disciplinary affiliation and high when lead authors come from a wide variety of disciplines—increased with time in all three communities (Fig 1B), but this was strongly correlated with the number of papers (S6 Fig). The best description of temporal dynamics in lead author diversity was a generalized additive model (GAM) that let each community follow its own trajectory (Akaike information criterion [AIC] for the saturated generalized linear model [GLM] = 48.33; AIC for GAM with common trajectory structure = 71.9; AIC for the GAM with varying trajectory structure = 21.5). That model explained 92.7% of observed deviation (coefficient estimates in S9 Table). Author diversity within papers also increased through time (S7A and S7B Fig and S8 Table) and was not strongly correlated with the number of authors on the paper (S6 Fig). However, both lead author diversity and author diversity within a paper were lower for the two traditional disciplines than for Group 3.

**Fig 2:**
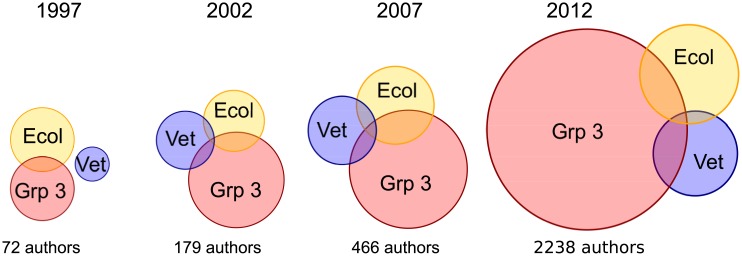
Venn diagrams of cross-community authorship through time. Each year’s Venn diagram is scaled to reflect the number of authors with two or more papers in our paper bank over the preceding 5 y. Number of authors with two papers in the same journal community are represented by disjointed regions of the circles, and number of authors with papers in two different communities are represented by the area of the intersections. Each circle is scaled to reflect the total number of authors with papers in that community during the 5 y prior to the label year. Areas are on a log-scale, and total number of authors with multiple papers each year is reported below each Venn diagram. Data to generate this figure are contained in S1 Data.

The number of authors contributing papers to two or more communities also increased with time (Fig 2). The proportion of authors with two or more papers who contributed papers to multiple communities also increased up until 2012, when it was likely halted by the rapid growth of Group 3.

### Journal Communities Varied in Study System, Question, and Analytical Approach

We read a random stratified sample of 236 papers designed to capture research trends in each journal community through time. Fifty-three (22.5%) of those papers did not include dynamic models of disease transmission and were therefore removed, leaving 183 papers: 50 from the ecology journal community, 64 from the veterinary journal community, and 69 from Group 3.

**Fig 3:**
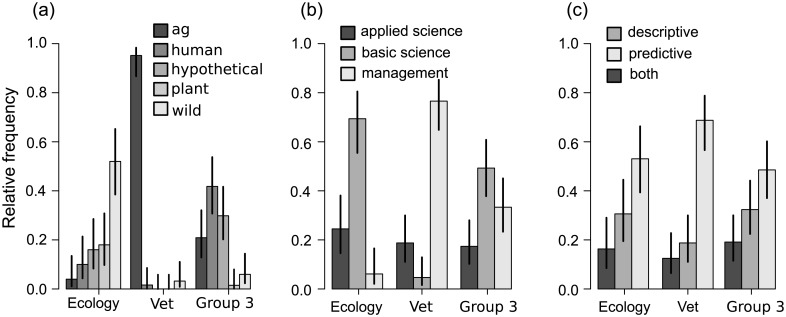
Model objectives from the three journal communities. (A) Study system: agricultural (domestic animals), human, hypothetical, plant, or wildlife. (B) Applied, basic science, or management objectives by community. “Applied science” was used to describe scenarios in which basic science questions were addressed using systems of management interest. (C) Predictive or descriptive modeling intent. Error bars depict 95% binomial confidence bounds. Data to generate this figure are contained in S2 Data.

Perhaps the most profound content difference among the three journal communities was the study system. Fifty-nine of the 64 manuscripts (92.2%) from the veterinary journal community focused on domestic animal systems; the ecological journals were dominated by studies of plant and wildlife systems (35 of 50 papers; 70.0%); and human systems were primarily studied in Group 3 (28 of 69 papers, 40.6% human) (Fig 3A). The communities also differed fundamentally in their approaches, objectives, and methodologies. Veterinary studies were more likely to be predictive and management-focused than studies in ecology or human-focused epidemiology (Fig 3B and 3C). Analyses aimed at gaining basic science insights were common in the ecological community and Group 3, but rare in the veterinary community (Fig 3B).

The veterinary community often included data in their analyses (S3 Fig), while this was not the case in the ecology community or Group 3; however, none of the communities regularly reported model performance of fit (S3 Fig). Simulation—both stochastic and deterministic—was widely used in all communities (S4 Fig), with a heavy emphasis on model sensitivity in the veterinary community (S4 Fig), and deterministic predictions based on mathematical analysis in the ecology community (S4 Fig).

### Publications Increasingly Cited Members of Their Own Community and Group 3

**Fig 4:**
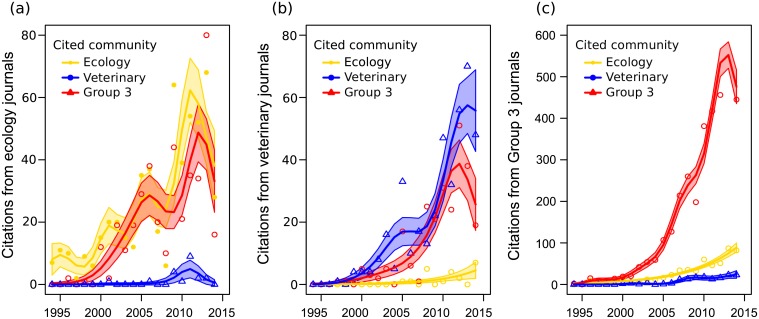
Cross-disciplinary citations through time. (A) Citations from papers in ecology journals to papers in each journal community. (B) Citations from papers in veterinary journals to papers in each journal community. (C) Citations from papers in Group 3 journals to papers in each journal community. Shaded regions are 95% confidence intervals from a Poisson generalized additive model fit to each journal community's time series. Data to generate this figure are contained in S1 Data.

All three communities cited other papers from their own community at increasing rates through time (Fig 4, S10 Table). Internal citation rates were highest for Group 3, due in part to its dramatically increasing size. Over that same period, the ecology and veterinary communities also increased the rate at which they cited Group 3, and Group 3 increased the rate at which it cited ecology parallel to ecology’s increase in internal citations. However, the ecology and veterinary communities did not increase citation rates toward one another, suggesting strong segregation in their respective bodies of literature. The apparent decline in recent citations (Fig 4) is consistent with other bibliometric analyses showing that citation distributions from papers published in a given year peak 3 y prior [18].

**Fig 5:**
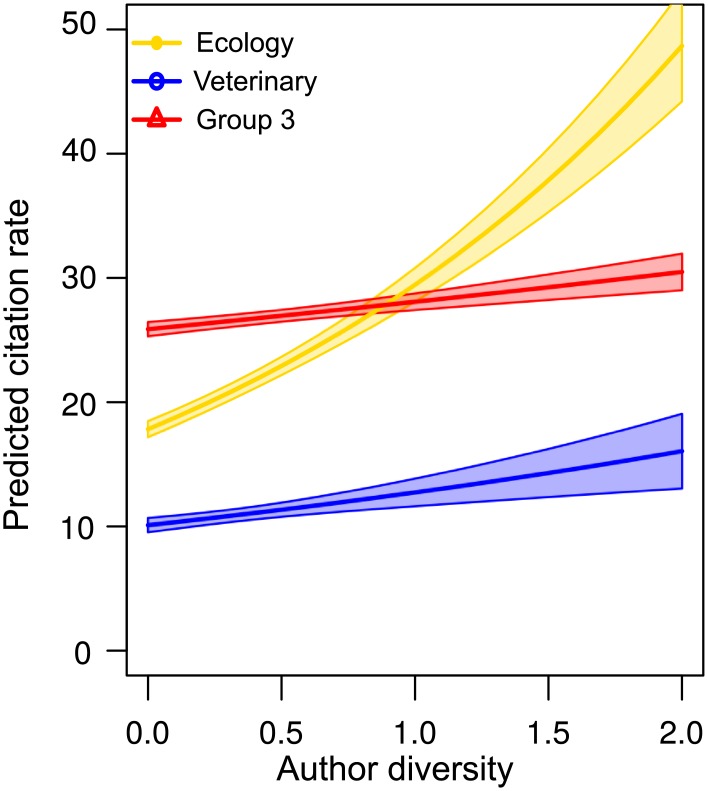
Citation benefits of author diversity. Associations between author diversity and citation rate for papers in each journal community. Model estimates are derived from a Poisson mixed effects model with an offset term for years since publication, and coefficient estimates are reported in S8 Table. Predictions are calculated for papers published in 2010, with 25% of citations to other journal communities and 75% of citations to the paper’s own community. Data to generate this figure are contained in S1 Data.

The effect of a focal paper’s author diversity on its citation rates—calculated as the number of papers within our search that cited the focal paper per year—varied between communities (Fig 5, S10 Table). In ecology, increased author diversity was associated with a substantial increase in a paper’s expected citation rate, but this effect was significantly lower for papers published in the veterinary community or Group 3.

## Discussion

We found three pieces of evidence suggesting the dynamic disease modeling community grew and diversified in tandem with increasing calls for One Health. First, the number of publications presenting dynamic infectious disease models grew rapidly relative to publication growth rates in the natural and health sciences as a whole (Fig 1B), indicating an intensifying research interest in this aspect of One Health. Second, the number of authors who contributed papers to journals in two or more journal communities also increased with time (Fig 2). Third, the fastest-growing community (Group 3) contained a broad and balanced range of contributing disciplines (Fig 1A) and captured the majority of papers fulfilling our search criteria (Fig 1B, S6 Table); we tentatively suggest this journal community could actually be labelled “One Health.”

Not all of our findings are so positive, however. Two traditional publication silos, ecology and veterinary medicine, remained segregated from one another. While system may be the nominal factor separating these disciplines (Fig 3A), their publications also partitioned along methodological and theoretical axes. All three communities ask unique questions (Fig 3B) and address those questions with unique methods (S3 and S4 Figs); we saw limited evidence of information movement between silos. Therefore, although our analysis uncovered an active interdisciplinary disease modeling publication forum, some fundamental challenges remain. These challenges are especially important for emerging infectious diseases with both domestic and wildlife hosts, since those systems are the purview of the segregated veterinary and ecological communities.

One additional observation merits mentioning: only three of the 1,628 papers included in this analysis derived from high-profile medical journals. Of the 25 highest-ranked medical journals in 2014, only *New England Journal of Medicine* and *PLoS Medicine* contained any papers fulfilling our search. While epidemiology journals were well represented in our survey, only 83 of the 6,450 ranked medical journals fall into that group. Limited medical representation in disease transmission studies (Fig 1A) poses an obstacle for strengthening the interface between human medicine and other facets of One Health.

Some continued disciplinary isolation is neither surprising nor problematic. Expertise on within-host processes, between-host processes, and mathematical modeling is the realm of distinct academic domains, and each domain requires intensive study on the way to expertise. Yet research teams that bridge traditional research silos are essential for efficient science, and building those teams is difficult. For example, a survey of researchers working on coupled human-natural systems (CHNs) documented tension between collaborating departments and institutions as a common hindrance to interdisciplinary work [19], and the majority of respondents in that study also reported difficulties in finding publication venues for interdisciplinary projects. Funding was cited as the primary obstacle to forming interdisciplinary collaborations, and researchers across participating disciplines reported limited credit for interdisciplinary work as a major barrier to interdisciplinarity [19]. In a different study of interdisciplinary geography faculty [20], participants expressed general scepticism toward researchers whose expertise differed from their own. If these sentiments also apply to researchers in One Health, they may form a barrier to future collaboration.

One Health training programs must provide students with sufficient depth in their own domain while also providing them with sufficient cross-disciplinary perspective to participate in multidisciplinary work. In traditional academic settings, graduate students are subject to expectations from their home departments, which may not coincide with an interdisciplinary team’s goals. Explicitly interdisciplinary graduate programs come with their own challenges. One study of 45 interdisciplinary neuroscience doctoral students [21] found that both students and faculty were dissatisfied with time constraints and imperative trade-offs of depth for breadth required by their interdisciplinary curriculum. Parallel sentiments arose among geography research faculty members [20] and graduate students from three different Integrative Graduate Education and Research Traineeship (IGERT) programs [22,23]. Interdisciplinary collegiate and graduate programs continue to grow in favor and number [24], however, so designing programs in the quantitative, ecological, veterinary, and medical sciences that balance discipline-specific and cross-disciplinary perspectives is a critical next step for One Health.

Researchers incur a cost—in visibility, productivity, and income—for failing to specialize [25]. One study found a significant positive relationship between specialization under a variety of metrics and productivity, and between productivity and income; researchers need strong incentives to overcome that cost and engage in cross-disciplinary collaborations. Horizontal funding structures (e.g., National Science Foundation [NSF]-National Institutes of Health [NIH]-US Department of Agriculture [USDA] Ecology and Evolution of Infectious Diseases [EEID]; Research and Policy for Infectious Disease Dynamics [RAPIDD]), training centers (e.g., the National Institute for Mathematical and Biological Synthesis [NIMBioS], Mathematical Biosciences Institute, Los Alamos National Laboratory’s Center for Nonlinear Studies), and academic consortia combat cost disincentives and form an active presence in today’s One Health community. Similar collaborations in the physical and medical sciences served as a basis for building interdisciplinary research teams [26] and increasing participant productivity [27]. Cross-disciplinary participant collaboration increased after attending workshops at NIMBioS [28], and similar, albeit undocumented, patterns probably surround many One Health gatherings.

In order to move forward, we must determine which projects intended to enhance One Health succeeded and identify factors that continue to impede interdisciplinarity. More information on the costs and benefits of different kinds of interactions (for example, participating in a common workshop, occupying a common institutional building, attending common seminars, and so on) would help clarify the most efficient means for building One Health teams.

One Health collaborations will likely continue increasing in frequency. Emerging quantitative methods like Bayesian hierarchical modeling, approximate Bayesian computation methods [29], and partially observed Markov models [30] offer new platforms for integrating data from veterinary studies, theoretical concepts from ecology, and detailed mechanistic models from mathematical epidemiology. Knowledge from captive or domestic animal systems may help researchers constrain some parameters in dynamic models of wild systems, and knowledge from disease transmission studies in wildlife may help refine captive study design. However, these benefits will not accumulate overnight.

Ideally, each One Health researcher should appreciate all aspects of field, modeling, and captive study, and interact with several of these components directly. Short of that goal, One Health requires, at a minimum, open-mindedness to varying approaches among individuals, training cultures, and publication venues. Funding agencies incentivized One Health collaborations by restructuring grants to favor interdisciplinary teams, and research institutions could do the same by restructuring tenure expectations to account for interdisciplinary effort (for example, by incorporating performance measures that account for cross-disciplinarity [31]). Editors in all contributing disciplines—but especially ecology and veterinary medicine—could help by accepting for review more disease papers that were historically perceived as too far afield. We see clear evidence of an emerging One Health community; however, without sustained efforts to integrate ecology, veterinary, and human medicine, disease modeling efforts will remain in some sense more “Three Healths” than “One.”

## Methods

We followed standard structured review protocols [32] to conduct a literature search for papers containing dynamic models [13] with nonlinear aspects of disease transmission (search terms and modifications are listed in S1–S3 Tables). We constructed three networks—one of papers, one of authors, and one of journals—to examine relationships in our paper bank. A walk-trap algorithm [15] with four steps was used to characterize structure in each network and to identify the number and composition of node communities. Community structure identified for the journal network formed the basis for our follow-up analyses. We classified text strings in author institute affiliations (for example, “vet,” “ecol,” “math”) to determine a discipline for each paper's lead author. Paper inclusion by era and discipline are shown in S4 Table.

We estimated the paper bank’s growth rate using a linear model. Let *P*_*t*_ be the number of papers returned by our search in year *t*. Then we assumed a Gaussian error structure and fit the model log(*P*_*t*_ + 1) = *β*_0_ + *β*_1_ *t* + *ε*. In this model, percent growth is estimated with the *β*_1_ term; approximate time to doubling is calculated as log (2)log (1+ β1). Due to the high number of zeros in the dataset prior to 1990, we fit this model on data from 1990 to present.

We read a stratified sample of 236 papers from the paper bank to compare modeling objectives, approaches, and data incorporation across journal communities over time, and by paper impact. Strata were defined by journal community, year of publication, and annual paper citation rate (total number of citations per year, divided by years since publication). In each sampled paper, the study system was classified as human, domestic animal, wildlife, plant, or hypothetical. Papers were identified as being predictive (using models to project future scenarios under varying conditions) or descriptive (using models to describe and gain insights from existing datasets), and were classified as primarily seeking insights about basic science, applied science (basic science questions addressed in a system of management or conservation importance), or management. Recorded fields are listed and defined in S12 Table. We identified highly cross-disciplinary journals by comparing the composition of lead author affiliations across all papers in our paper bank to the composition of lead author affiliations within paper bank papers from each particular journal.

We used GAMs to describe two measures of author diversity and community-to-community citation rates through time. We used Shannon’s diversity index, *H*′, a metric that accounts for both number and relatively frequency of different unit types, to quantify author affiliation diversity. Under Shannon’s diversity, a community with *I* distinct categories, each represented at proportion *p*_*i*_, is assigned the value $H^{'}\;=\;\sum_{i\;=\;1}^{I}p_{i}log(p_{i})$. We measured author diversity within papers (*H*′_*C*_) and lead author diversity within communities (*H*′_*L*_) and modeled each as a function of journal community and year. Saturated models included a community-specific intercept *α*_*j*_, a spline function describing changes over years (*s*_1_), and a spline term capturing interaction between year and community (*s*_2_), where community is denoted by indicator variables *Comm*_1_,*Comm*_2_, and *Comm*_3_. For the *i* ^*th*^ observation (${{H'}_{L}}_{i}$ and ${{H'}_{C}}_{i}$) in journal community *j*, this model can be written as

$${H'}_{i}\;=\;\alpha_{j\left[i\right]}+s_{1}({Year}_{i})+s_{2}({Year}_{i})\times {Comm}_{j\left[i\right]}+\varepsilon_{i},$$

where *ε*_*i*_ ~ *N*(0,*σ* ^2^). Community-to-community citation rates were modeled using the count of papers published in community *k* that were cited by papers published in community *l*. Counts were treated as Poisson, and model covariate structure was identical to the author diversity models described previously. Saturated models were reduced in accordance with AIC.

We identified factors leading to high citation rates by modeling how many times each paper was cited (denoted *Y*_*i*_, *Y*_*i*_ ~ *Poisson*(*λ*_*i*_)) as a function of that paper's within-paper authorship diversity (*H*′_*C*_), citation diversity (*R*, the ratio of between-community citations to total citations), and publication year (*Year*). The model allowed for community-specific intercepts (*α*_*j*[*i*]_), slopes associated with author diversity (*δ*_*j*[*i*]_), and slopes associated with citation diversity (*ϕ*_*j*[*i*]_). The model had a log-link and an offset term for years since publication, and can be written as

$$\lambda_{i}\;=\;exp\;\left[\alpha_{j\left[i\right]}+\beta {Year}_{i}+\delta_{j\left[i\right]}{{H'}_{C}}_{i}+\phi_{j\left[i\right]}R_{i}+\varepsilon_{j}\right].$$

A complete description of the literature search, network construction, stratified sampling, and analysis is included in S1 Text.

## Supporting Information

S1 Data Full paper bank data. A csv file containing bibliographic information on all papers fulfilling our search criteria. This dataset was used to construct Figs 1, 2, 4 and 5.
(CSV)

S2 Data Data from sampled papers. A csv file containing all data extracted from the papers we read in our detailed sample. These data were used to construct Fig 3.
(CSV)

S3 Data Comparison of community classifications. A csv file containing community assignments for journals configured in an undirected network under three different community detection algorithms (fast-greedy, spin-glass, and Louvain detection). Note that the classifications used in the main text are listed in S1 Text.
(CSV)

S1 Fig The journal network, color-coded by journal community membership. Journals assigned by the walk-trap algorithm to the ecology community are in gold ("Ecol"); journals in the veterinary community are in blue ("Vet"); journals in "Group 3" are in red; and green nodes reflect outlying journals that were not explicitly assigned to any one community. Data to generate this figure are included in S2 Data.
(PNG)

S2 Fig Journal community structure. (A) Within- and between-journal-community citation frequencies. (B) Edge width scales with the number of cross-community citations. Node size scales with the number of papers from each community included in our paper bank (ranging from 198 in the veterinary community to 1,043 in Group 3). Data to generate this figure are included in S2 Data.
(PNG)

S3 Fig Model testing and evaluation patterns. (A) Data incorporation by community. (B) Incorporation of new data in modeling papers by community. (C) Assessment of model fit by community. Error bars show 95% binomial confidence limits. Data to generate this figure are included in S3 Data.
(PNG)

S4 Fig Modeling approaches across communities. (A) Stochastic or deterministic modeling approaches; (B) Papers incorporating simulation; (C) Papers incorporating sensitivity analyses; (D) Papers incorporating other methods of mathematical evaluation including mathematical proof, equilibrium analyses, derivation of new theoretical relationships, asymptotic conditions, etc. Error bars depict 95% binomial confidence bounds. Data to generate this figure are included in S3 Data.
(PNG)

S5 Fig Number of papers and journals returned by our search through time. The left panel shows papers and journals summed across all journal communities; the right panel shows papers and journals within each community through time. Data to generate this figure are included in S2 Data.
(PDF)

S6 Fig Diversity by number of units (authors or papers). The left panel shows within-paper author diversity as a function of number of authors on that paper for all papers in our paper bank. The right panel shows lead author diversity within a community as a function of the number of papers in that community (the plot consists of one point for each journal community in each year from 1995 to 2014). Data to generate this figure are included in S2 Data.
(PNG)

S7 Fig Temporal dynamics of author diversity. (A) Author domain diversity (measured using Shannon's diversity index, H’) within papers through time in each community. (B) Proportion of papers with author diversity equal to zero through time in each journal community. Data to generate this figure are included in S2 Data.
(PDF)

S1 Table Search terms, term content, and term objectives. (DOCX)

S2 Table SciImago journal categories included. (DOCX)

S3 Table Added journals. Journals added to initial search because they were cited 250 or more times by papers returned in the initial search of highly ranked journals.
(DOCX)

S4 Table Number of papers in the three dominant journal communities through time. (DOCX)

S5 Table Descriptive metrics from the co-author, paper, and journal networks. Nodes are the number of entities in the network, and edges represent co-authors (Authors), cited papers (Papers), and citations between journals (Journals). “Undirected” networks have symmetric edges linking nodes (e.g., network has an identical strength connecting node A to B and node B to A). Size of the largest component is measured in number of nodes. Isolated nodes are completely unconnected from the rest of the network. Mean degree is the average number of edges connected to each node, diameter is the maximum distance between any pair of connected nodes, and mean path length is the average minimum distance between any pair of connected nodes.
(DOCX)

S6 Table Journal community attributes from the three major journal communities. “Directed” networks are those in which edges need not be symmetric in weight (i.e., one paper can cite another without the reciprocal occurring, so the paper network is directed; co-authorship roles, however, are reciprocal, so the author network is undirected). Lead author affiliation was designated based on author institution as listed in the paper bank metadata. Total and percent citations from papers in each community to papers in the other communities are shown in the final three columns. Within-community citations are bolded.
(DOCX)

S7 Table Publication growth rate model output. (DOCX)

S8 Table Model output from the within-paper author diversity GAM. (DOCX)

S9 Table Model output from the lead author diversity GAM. (DOCX)

S10 Table Model output from the citation rate GAM. (DOCX)

S11 Table GLM output for the citation rate model. (DOCX)

S12 Table Fields collected on all 236 papers that were read in full. Possible responses in parentheses; options separated by /.
(DOCX)

S1 Text Additional methods. Detailed descriptions of data extraction protocols and analyses, as well as supporting results, including tables and figures that complement the main text.
(DOCX)

## Abbreviations

AIC: Akaike information criterion
CHN: coupled human-natural system
EEID: Ecology and Evolution of Infectious Diseases
EID: emerging infectious disease
GAM: generalized additive model
GLM: generalized linear model
IGERT: Integrative Graduate Education and Research Traineeship
NIH: National Institutes of Health
NIMBioS: National Institute for Mathematical and Biological Synthesis
NSF: National Science Foundation
RAPIDD: Research and Policy for Infectious Disease Dynamics
USDA: United States Department of Agriculture
