# Supplementary figures and images for: “One Health” or Three? Publication Silos Among the One Health Disciplines
Source: PLoS Biol. 2016 Apr 21;14(4):e1002448. doi: 10.1371/journal.pbio.1002448 (PMC4839662; doi:10.1371/journal.pbio.1002448)

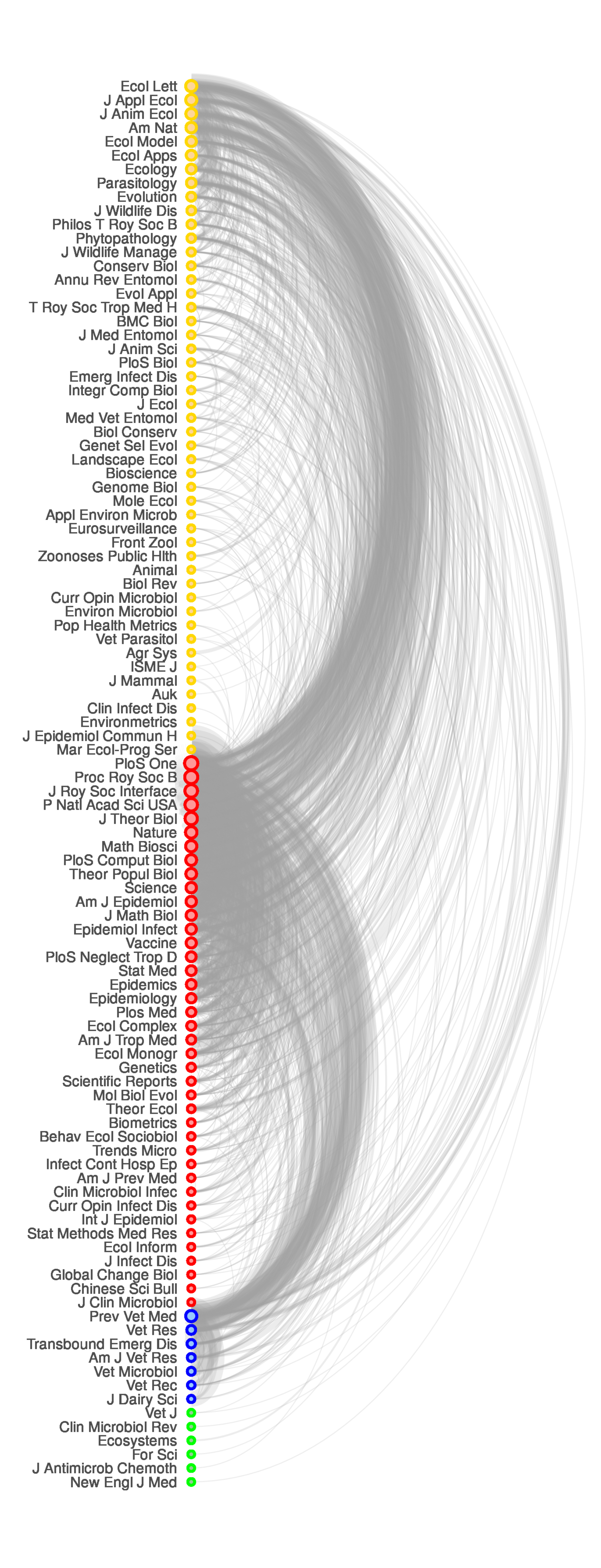

Supplement: S1 Fig — Journals assigned by the walk-trap algorithm to the ecology community are in gold ("Ecol"); journals in the veterinary community are in blue ("Vet"); journals in "Group 3" are in red; and green nodes reflect outlying journals that were not explicitly assigned to any one community. Data to generate this figure are included in S2 Data. (PNG) [file pbio.1002448.s004.png]

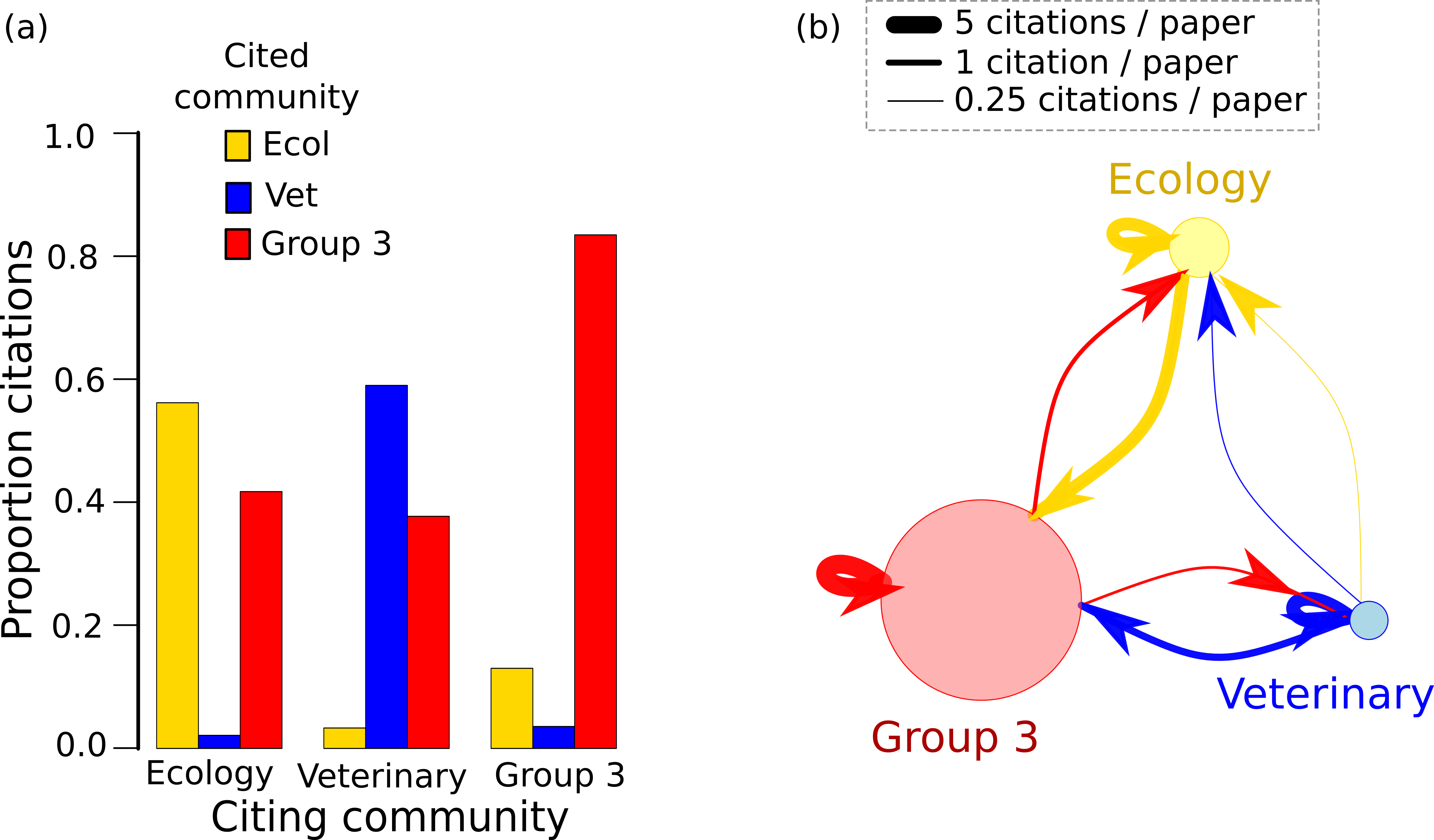

Supplement: S2 Fig — (A) Within- and between-journal-community citation frequencies. (B) Edge width scales with the number of cross-community citations. Node size scales with the number of papers from each community included in our paper bank (ranging from 198 in the veterinary community to 1,043 in Group 3). Data to generate this figure are included in S2 Data. (PNG) [file pbio.1002448.s005.png]

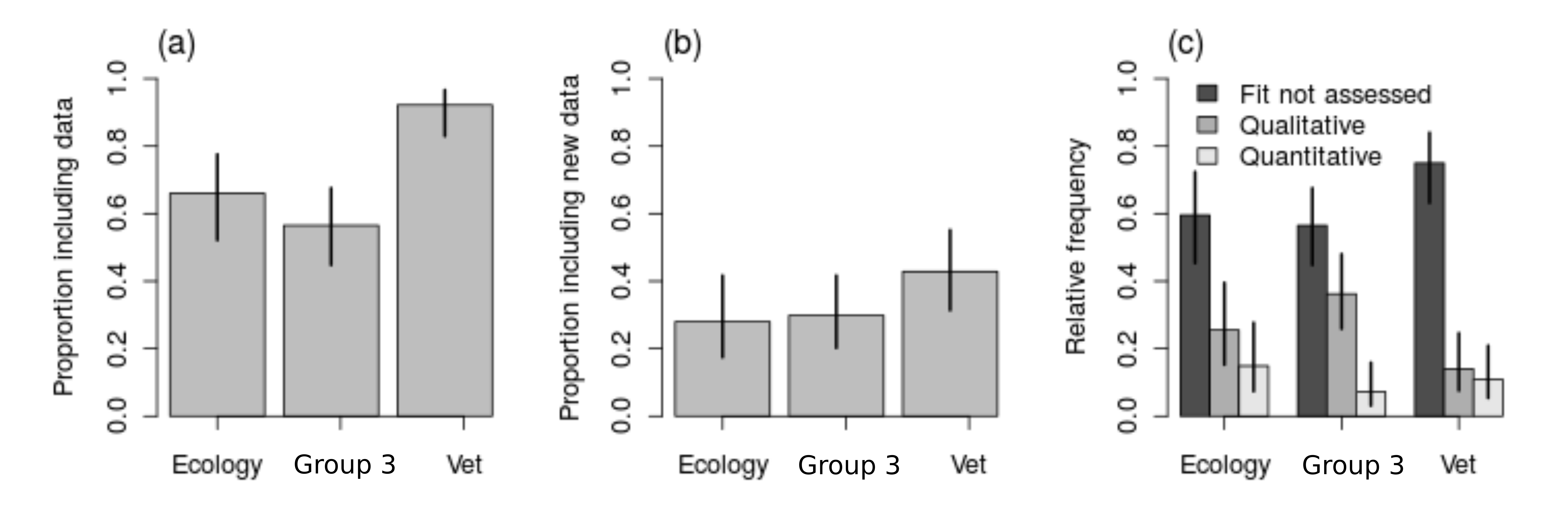

Supplement: S3 Fig — (A) Data incorporation by community. (B) Incorporation of new data in modeling papers by community. (C) Assessment of model fit by community. Error bars show 95% binomial confidence limits. Data to generate this figure are included in S3 Data. (PNG) [file pbio.1002448.s006.png]

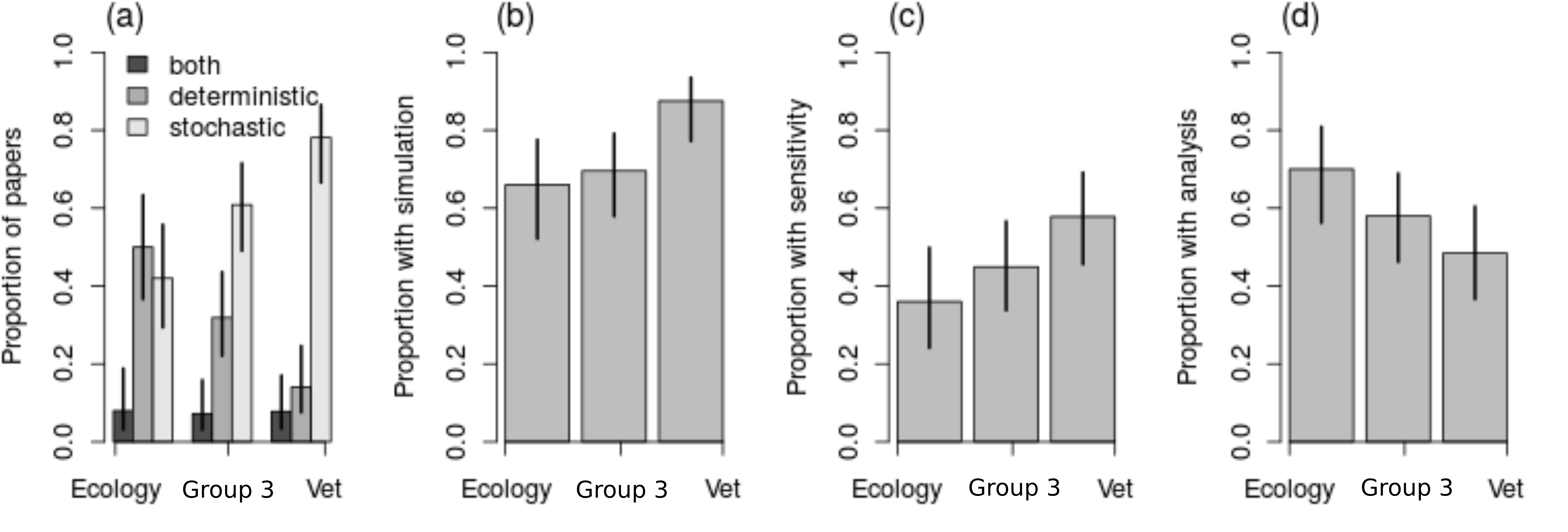

Supplement: S4 Fig — (A) Stochastic or deterministic modeling approaches; (B) Papers incorporating simulation; (C) Papers incorporating sensitivity analyses; (D) Papers incorporating other methods of mathematical evaluation including mathematical proof, equilibrium analyses, derivation of new theoretical relationships, asymptotic conditions, etc. Error bars depict 95% binomial confidence bounds. Data to generate this figure are included in S3 Data. (PNG) [file pbio.1002448.s007.png]

Number included in search

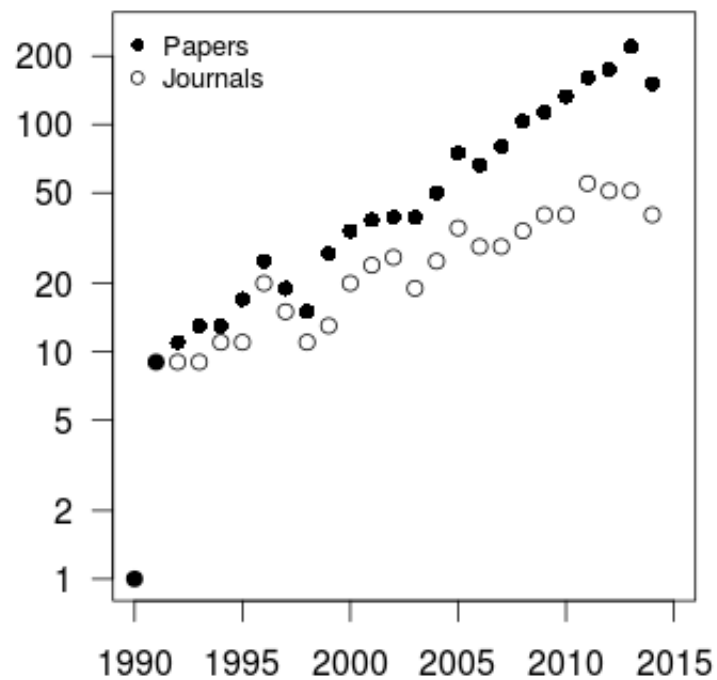

Number included in search

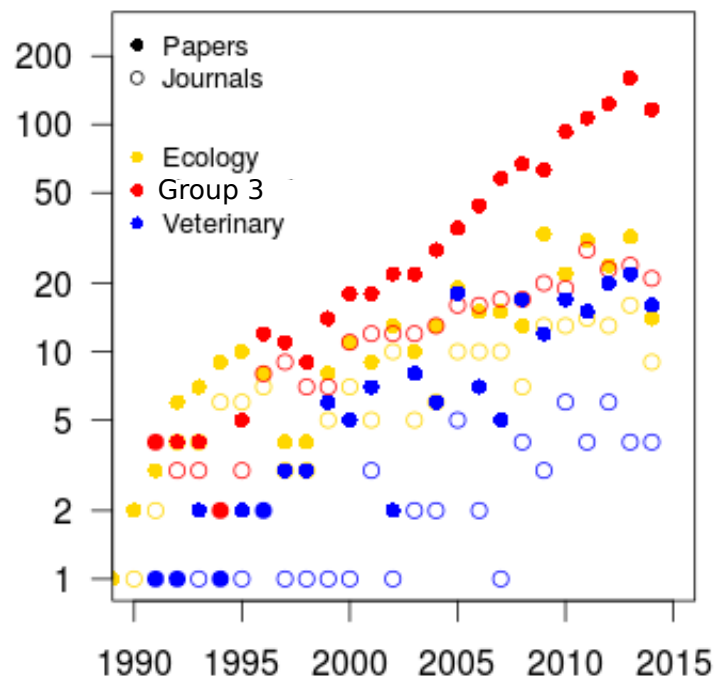

Supplement: S5 Fig — The left panel shows papers and journals summed across all journal communities; the right panel shows papers and journals within each community through time. Data to generate this figure are included in S2 Data. (PDF) [file pbio.1002448.s008.pdf]

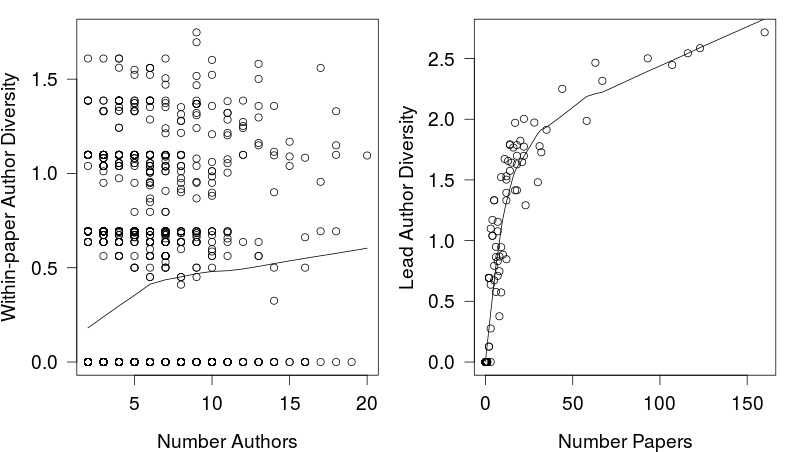

Supplement: S6 Fig — The left panel shows within-paper author diversity as a function of number of authors on that paper for all papers in our paper bank. The right panel shows lead author diversity within a community as a function of the number of papers in that community (the plot consists of one point for each journal community in each year from 1995 to 2014). Data to generate this figure are included in S2 Data. (PNG) [file pbio.1002448.s009.png]

Author diversity within papers

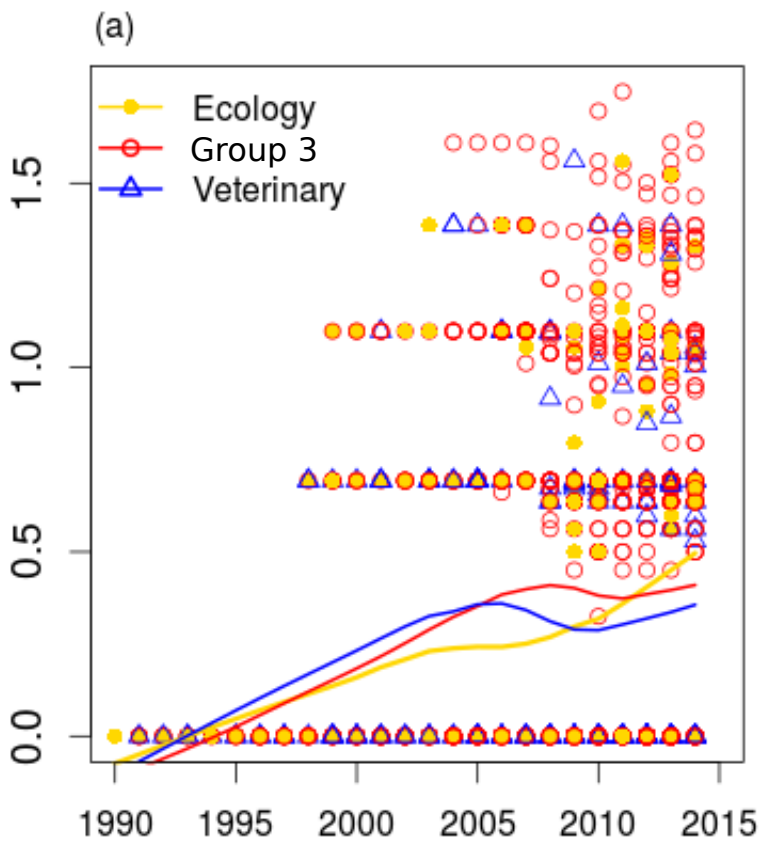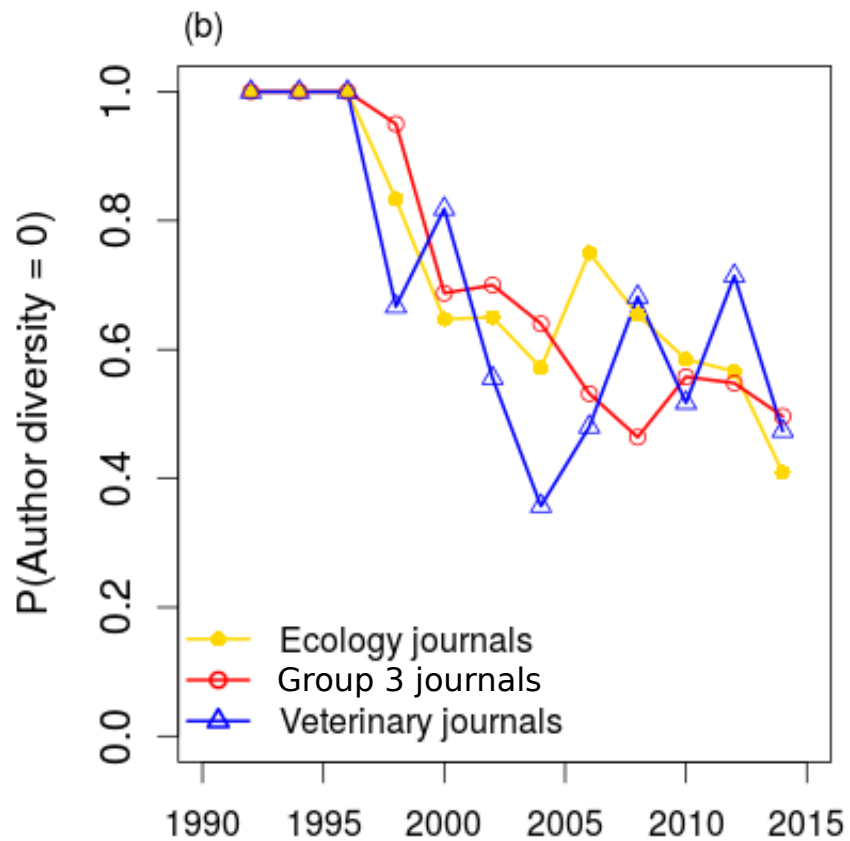

Supplement: S7 Fig — (A) Author domain diversity (measured using Shannon's diversity index, H’) within papers through time in each community. (B) Proportion of papers with author diversity equal to zero through time in each journal community. Data to generate this figure are included in S2 Data. (PDF) [file pbio.1002448.s010.pdf]
