# Supplementary material for: “One Health” or Three? Publication Silos Among the One Health Disciplines
Source: PLoS Biol. 2016 Apr 21;14(4):e1002448. doi: 10.1371/journal.pbio.1002448 (PMC4839662; doi:10.1371/journal.pbio.1002448)
Supplement: S1 Table — (DOCX) [file pbio.1002448.s011.docx]

**S1 Table. Search terms, term content, and term objectives.**

| **Name** | **Content** | **Objective** |
| --- | --- | --- |
| T1 | (disease AND propogat*) OR (disease AND persist*) OR (disease AND infect*) OR (disease AND transmi*) OR (disease AND force of infect*) OR (disease AND R0) OR (disease AND R-naught) OR (disease AND R-0) | Capture references to disease transmission |
| T2 | AND (dynami* OR simulat*) | Limit to papers emphasizing dynamic process |
| T3 | AND (mechanis* OR stochastic* OR determinis* OR compartment* OR sensitivit* OR statistic* OR estimat*) | Limit to papers emphasizing dynamic process |
| T4 | NOT (mouse model OR protease OR drug-drug interaction OR neutraliz* OR inhibitor* OR unfold* OR variant OR homolog* OR "in vivo" OR "in vitro" OR CD4 OR deep sequenc* OR enzyme OR "infected cell*") | Eliminate within-host models |
| T5 | NOT (HIV OR AIDS OR human immunodeficiency virus) | Eliminate HIV/AIDS models |
