# Supplementary material for: “One Health” or Three? Publication Silos Among the One Health Disciplines
Source: PLoS Biol. 2016 Apr 21;14(4):e1002448. doi: 10.1371/journal.pbio.1002448 (PMC4839662; doi:10.1371/journal.pbio.1002448)
Supplement: S2 Table — (DOCX) [file pbio.1002448.s012.docx]

**S2 Table. SciImago journal categories included.**

| **SciMago General Category** | **Subcategory** |
| --- | --- |
| Agricultural and Biological Sciences | Animal Science and Zoology |
|  | EEBS |
|  | Agricultural and Biological Sciences Miscellaneous |
| Environmental Science | Ecological Modeling |
|  | Ecology |
|  | Nature and Landscape Conservation |
| Medicine | Epidemiology |
|  | Infectious Disease |
| Veterinary | All Categories |
