# Supplementary material for: “One Health” or Three? Publication Silos Among the One Health Disciplines
Source: PLoS Biol. 2016 Apr 21;14(4):e1002448. doi: 10.1371/journal.pbio.1002448 (PMC4839662; doi:10.1371/journal.pbio.1002448)
Supplement: S3 Table — Journals added to initial search because they were cited 250 or more times by papers returned in the initial search of highly ranked journals. (DOCX) [file pbio.1002448.s013.docx]

**S3 Table. Added journals.** Journals added to initial search because they were cited 250 or more times by papers returned in the initial search of highly ranked journals.

| American Journal of Human Genetics |
| --- |
| American Journal of Tropical Medicine and Hygiene |
| Epidemiology and Infection |
| Evolution |
| Genetics |
| Journal of Clinical Microbiology |
| Journal of the Royal Society Interface |
| Journal of Theoretical Biology |
| Journal of Virology |
| Journal of Wildlife Diseases |
| Mathematical Biosciences |
| New England Journal of Medicine |
| Phytopathology |
| PLoS Medicine |
| Theoretical population biology |
| Transactions of the Royal Society of Tropical Medicine and Hygiene |
| Veterinary Record |
