# Supplementary material for: “One Health” or Three? Publication Silos Among the One Health Disciplines
Source: PLoS Biol. 2016 Apr 21;14(4):e1002448. doi: 10.1371/journal.pbio.1002448 (PMC4839662; doi:10.1371/journal.pbio.1002448)
Supplement: S4 Table — (DOCX) [file pbio.1002448.s014.docx]

**S4 Table. Number of papers in the three dominant journal communities through time.**

|  | **Biological journals** | **Ecological journals** | **Vet journals** | **Total** |
| --- | --- | --- | --- | --- |
| **1998-2002** | 81 | 40 | 23 | 144 |
| **2003-2007** | 188 | 67 | 44 | 299 |
| **2008-2012** | 453 | 118 | 81 | 652 |
| **Total** | 1043 | 310 | 198 |  |
