# Supplementary material for: “One Health” or Three? Publication Silos Among the One Health Disciplines
Source: PLoS Biol. 2016 Apr 21;14(4):e1002448. doi: 10.1371/journal.pbio.1002448 (PMC4839662; doi:10.1371/journal.pbio.1002448)
Supplement: S5 Table — Nodes are the number of entities in the network, and edges represent co-authors (Authors), cited papers (Papers), and citations between journals (Journals). “Undirected” networks have symmetric edges linking nodes (e.g., network has an identical strength connecting node A to B and node B to A). Size of the largest component is measured in number of nodes. Isolated nodes are completely unconnected from the rest of the network. Mean degree is the average number of edges connected to each node, diameter is the maximum distance between any pair of connected nodes, and mean path length is the average minimum distance between any pair of connected nodes. (DOCX) [file pbio.1002448.s015.docx]

**S5 Table. Descriptive metrics from the coauthor, paper, and journal networks.** Nodes are the number of entities in the network and edges represent coauthors (Authors) cited papers (Papers) and citations between journals (Journals). “Undirected” networks have symmetric edges linking nodes (e.g., network has an identical strength connecting node A to B and node B to A). Size of the largest component is measured in number of nodes. Isolated nodes are completely unconnected from the rest of the network. Mean degree is the average number of edges connected to each node, diameter is the maximum distance between any pair of connected nodes, and mean path length is the average minimum distance between any pair of connected nodes.

| **Network** | **Number nodes/ edges** | **Directed / Undirected** | **Size largest component (2^nd^ largest)** | **Number isolated nodes (%)** | **Mean Degree** | **Diameter** | **Mean path length** |
| --- | --- | --- | --- | --- | --- | --- | --- |
| Authors | 4309 / 16843 | Undirected | 3021 (19) | 22 (0.5%) | 7.98 | 15 | 5.87 |
| Papers | 1628/ 3492 | Directed | 1204 (6) | 347 (21.3%) | 2.17 | 8 | 3.01 |
| Journals | 108 / 840 | Directed | 95 (1) | 13 (12.0%) | 8.84 | 9 | 2.29 |

_
