# Supplementary material for: “One Health” or Three? Publication Silos Among the One Health Disciplines
Source: PLoS Biol. 2016 Apr 21;14(4):e1002448. doi: 10.1371/journal.pbio.1002448 (PMC4839662; doi:10.1371/journal.pbio.1002448)
Supplement: S6 Table — “Directed” networks are those in which edges need not be symmetric in weight (i.e., one paper can cite another without the reciprocal occurring, so the paper network is directed; co-authorship roles, however, are reciprocal, so the author network is undirected). Lead author affiliation was designated based on author institution as listed in the paper bank metadata. Total and percent citations from papers in each community to papers in the other communities are shown in the final three columns. Within-community citations are bolded. (DOCX) [file pbio.1002448.s016.docx]

**S6 Table. Journal community attributes from the three major journal communities.** “Directed” networks are those in which edges need not be symmetric in weight (i.e., one paper can cite another without the reciprocal occurring, so the paper network is directed; co-authorship roles, however, are reciprocal, so the author network is undirected). Lead author affiliation was designated based on author institution as listed in the paper bank metadata. Total and percent citations from papers in each community to papers in the other communities are shown in the final three columns. Within-community citations are bolded.

| **Community** | **Number of Journals** | **Number of Papers** | **Median Number of Authors (2.5th, 97.5^th^ quantiles)** | **Most common lead author affiliation (%)** | **Citations to human- focused epi** | **Citations to ecology** | **Citations**  **to veterinary** |
| --- | --- | --- | --- | --- | --- | --- | --- |
| Human-focused Epidemiology | 42 | 1043 | 4 (1, 15) | math / stat / epi (48.2%) | **3488 (84.4%)** | 393 (9.5%) | 250 (6.1%) |
| Ecology | 30 | 310 | 4 (1, 12.275) | ecology / evolution (55.9%) | 544 (49.7%) | **529 (48.3%)** | 22 (2.0%) |
| Veterinary | 7 | 198 | 4 (1, 9.075) | veterinary / animal health (63.6%) | 149 (26.6%) | 20 (3.6%) | **391 / 169 (69.8%)** |
