# Supplementary material for: “One Health” or Three? Publication Silos Among the One Health Disciplines
Source: PLoS Biol. 2016 Apr 21;14(4):e1002448. doi: 10.1371/journal.pbio.1002448 (PMC4839662; doi:10.1371/journal.pbio.1002448)
Supplement: S7 Table — (DOCX) [file pbio.1002448.s017.docx]

**S7 Table. Publication growth rate model output.**

| **Variable** | **Estimate** | **Standard Error** | **t-value (p)** |
| --- | --- | --- | --- |
| Intercept | 1.790 | 0.134 | 13.38 (<0.0001) |
| Year | 0.146 | 0.008 | 1.31 (p<0.0001) |
