# Supplementary material for: “One Health” or Three? Publication Silos Among the One Health Disciplines
Source: PLoS Biol. 2016 Apr 21;14(4):e1002448. doi: 10.1371/journal.pbio.1002448 (PMC4839662; doi:10.1371/journal.pbio.1002448)
Supplement: S8 Table — (DOCX) [file pbio.1002448.s018.docx]

**S8 Table. Model output from the within-paper author diversity GAM.** .

| **Parametric Terms** | | | |
| --- | --- | --- | --- |
| **Variable** | **Estimate** | **Standard Error** | **t-value (p)** |
| Intercept (Ecology) | 0.358 | 0.027 | 13.02 (<0.001) |
| Human Epidemiology | 0.020 | 0.031 | 0.637 (0.525) |
| Veterinary | -0.217 | 0.043 | -0.505 (0.614) |
|  |  |  |  |
| **Smooth Terms** | | | |
| **Variable** | **Estimated degrees of freedom** | **Reference degrees of freedom** | **F (p)** |
| S(Year) | 4.070 | 5.101 | 4.15 (<0.001) |
| S(Year x Ecology) | 2.315 | 2.944 | 1.63 (0.181) |
| S(Year x Human epi) | 0.750 | 0.750 | 0.05 (0.853) |
| S(Year x Veterinary) | 0.750 | 0.750 | 0.43 (0.569) |

Deviance explained = 5.65%
