# Supplementary material for: “One Health” or Three? Publication Silos Among the One Health Disciplines
Source: PLoS Biol. 2016 Apr 21;14(4):e1002448. doi: 10.1371/journal.pbio.1002448 (PMC4839662; doi:10.1371/journal.pbio.1002448)
Supplement: S9 Table — (DOCX) [file pbio.1002448.s019.docx]

**S9 Table. Model output from the lead author diversity GAM.**

| **Parametric Terms** | | | |
| --- | --- | --- | --- |
| **Variable** | **Estimate** | **Standard Error** | **t-value (p)** |
| Intercept (Ecology) | 0.785 | 0.041 | 19.00 (<0.001) |
| Human Epidemiology | 0.490 | 0.058 | 8.38 (<0.001) |
| Veterinary | -0.172 | 0.058 | -2.94 (0.004) |
|  |  |  |  |
| **Smooth Terms** | | | |
| **Variable** | **Estimated degrees of freedom** | **Reference degrees of freedom** | **F (p)** |
| S(Year) | 2.518 | 3.181 | 6.18 (0.001) |
| S(Year x Ecology) | 5.978 | 7.121 | 2.28 (0.034) |
| S(Year x Human epi) | 4.344 | 5.373 | 5.45 (<0.001) |
| S(Year x Veterinary) | 0.750 | 0.750 | 2.06 (0.217) |

Deviance explained = 92.6%
