# Supplementary material for: “One Health” or Three? Publication Silos Among the One Health Disciplines
Source: PLoS Biol. 2016 Apr 21;14(4):e1002448. doi: 10.1371/journal.pbio.1002448 (PMC4839662; doi:10.1371/journal.pbio.1002448)
Supplement: S10 Table — (DOCX) [file pbio.1002448.s020.docx]

**S10 Table. Model output from the citation rate GAM.**

|  | **Smooth Terms** | | | |
| --- | --- | --- | --- | --- |
| **Variable** | | **Estimated degrees of freedom** | **Reference degrees of freedom** | **X-squared (p)** |
|  | S(Year) | 4.98 | 5.54 | 48.92 (<0.0001) |
| Citing community |  |  |  |  |
|  | S(Year x Ecology) | 5.09 | 5.62 | 59.92 (<0.0001) |
|  | S(Year x Human epi) | 6.18 | 6.62 | 86.53 (<0.0001) |
|  | S(Year x Veterinary) | 1.01 | 1.01 | 0.913 (.341) |
| Cited community |  |  |  |  |
|  | S(Year x Ecology) | 3.66 | 4.36 | 28.86 (<0.0001) |
|  | S(Year x Human epi) | 1.02 | 1.02 | 2.05 (0.156) |
|  | S(Year x Veterinary) | 6.05 | 6.62 | 119.08 (<0.001) |

Deviance explained = 88.3%
