# Supplementary material for: “One Health” or Three? Publication Silos Among the One Health Disciplines
Source: PLoS Biol. 2016 Apr 21;14(4):e1002448. doi: 10.1371/journal.pbio.1002448 (PMC4839662; doi:10.1371/journal.pbio.1002448)
Supplement: S11 Table — (DOCX) [file pbio.1002448.s021.docx]

**S11 Table. GLM output for the citation rate model.**

| **Variable** | **Estimate** | **Standard Error** | **z-value (p)** |
| --- | --- | --- | --- |
| ${H'}_{C}$ (Ecology) | -1851 | 1.945 | -951.5 (<0.0001) |
| Human Epi effect | 0.502 | 0.027 | 18.77 (<0.0001) |
| Veterinary effect | 0.396 | 0.023 | 17.45 (<0.0001) |
| Publication Year | 0.920 | 0.0009 | 945.64 (<0.0001) |
| *R* | 0.049 | 0.037 | 1.467 (0.142) |
| ${H'}_{C}\times$ Human Epi | -0.421 | 0.030 | -14.155 (<0.0001) |
| ${H'}_{C}\times$ Veterinary | -0.271 | 0.061 | -4.466 (<0.0001) |
| $R\times$ Human Epi | -0.091 | 0.043 | -2.134 (0.0329) |
| $R\times$ Veterinary | -0.473 | 0.076 | -6.192 (<0.0001) |
