# Supplementary material for: “One Health” or Three? Publication Silos Among the One Health Disciplines
Source: PLoS Biol. 2016 Apr 21;14(4):e1002448. doi: 10.1371/journal.pbio.1002448 (PMC4839662; doi:10.1371/journal.pbio.1002448)
Supplement: S12 Table — Possible responses in parentheses; options separated by /. (DOCX) [file pbio.1002448.s022.docx]

**S12 Table. Fields collected on all 236 papers that were read in full.** Possible responses in parentheses; options separated by /.

| **Col** | **Field Name** | **Description** |
| --- | --- | --- |
| A | PaperNumber | Position of paper in Web of Science search resuls; used to uniquely identify papers |
| B | Authors | All authors listed in manuscript’s Web of Science reference |
| C | Title | Paper’s title |
| D | Source | Journal where paper was published |
| E | Keep | Paper contained a direct or indirect reference to “disease transmission” in its title or abstract |
| F | BetweenHostTransmissionReferencedInAbstract | Is between-host transmission referenced directly or indirectly in abstract? (yes/no) |
| G | Review | Is the paper a review? (yes/no) |
| H | CertainPaperIncluded | Does paper contain dynamic model of pathogen transmission (e.g., does it contain a quantititative model of transmission? Is model nonlinear?)? (NA/0 = No / 1 = Yes / 2 = Ambiguous) |
| I | Pathogen | What is the focal pathogen? (Free text) |
| J | Host | What is the focal host species? (Free text) |
| K | Vector | What is the vector species? (Free text) |
| L | HumanAgWildPlant | Is this paper focused on human/ag/wildlife aspects of disease transmission? (“human”, “ag”, “wildlife”, “multiple”, “hypothetical”) |
| 25 | PredictiveDescriptive | Is model predictive / descriptive / both? (“predictive”, “descriptive”, “both”) |
| N | ModelObjective | What are the model objectives? (“management -- epidemic size” / “management -- vaccination” / “management -- surveillance” / “management -- economics” / “management -- eradication” / “management -- population viability” / “management -- epidemic control” / “basic science -- disease” / “basic science -- evolution” (e.g., testing hypotheses on a system with limited management interest) / “basic science -- theoretical relationships“ /“basic science -- estimation“ /“applied science” (e.g., addressing basic science questions on a system with management interest) / “VectorPopulationDynamics”) |
| O | DataIncorporated | Are data incorporated? (1 = yes / 0 = no) |
| P | ParamValsFromLit | Does the analysis use previously published empirical parameter estimates? (1 = yes / 0 = no) |
| Q | NewEmpiricalData | Does the paper contain previously unpublished data? (1 = yes / 0 = no). If paper references a particular person with unpublished data, this field takes on value 1. |
| R | FitAssessed | Is model fit assessed e.g., with Bayes factors/goodness-of-fit-like tests/posterior predictive checks? (1 = yes quantitative / 2 = yes qualitative / 3 = no) |
| S | NumberParams | On average, how many parameters does each model include? (integer) |
| T | CertainNumberParams | Are you certain about the number of parameters included in the model (0 = not at all certain / 1 = very certain / 2 = moderately certain (e.g., within 3 parameters)) |
| U | NumberModels | How many models were included in the paper? (integer) |
| V | ModelStructure | What model structure is used? (free text; see options below table*) |
| W | MathAssessment | Is an analytical/numerical assessment included? (1 = yes / 0 = no) |
| X | SensitivityAnalysis | Does the paper contain a sensitivity analysis? (1 = yes / 0 = no) |
| Y | Simulation | Is simulation included? (1 = yes / 0 = no) |
| Z | StochDeter | Are results based on stochastic models / deterministic models / both? (1 = yes) |
| AA | HeterogeneousStates | Include heterogeneity in disease state durations/intensities? (1 = yes / 0 = no) |
| AB | HeteroContactsSpat | Include heterogeneity in contact structure through metapopulation/spatial structure (1 = yes / 0 = no) |
| AC | HeteroContactsSocial | Include heterogeneity in contact structure through social network? (1 = yes) |
| AD | DataHostAbundance | Does the paper incorporate data on host abundance? (“empirical” /“simulated” / “no”) |
| AE | DataHostMovement | Does the paper incorporate data on host movement patterns (“empirical”/ “simulated” / “no”) |
| AF | DataPathGenetics | Does the paper incorporate data on pathogen genetics (“empirical” / “simulated” / “no”) |
| AG | DataDiseaseIncidence | Does the paper incorporate data on disease incidence/serology (“empirical” / “simulated” / “no”) |
| AH | ExpertOpinionUsed | Was expert opinion used in PARAMETERIZING the model(s)? (NA = Not applicable / 0 = no / 1 = yes / 2 = ambiguous) |
| AI | NHostIndivids | Number of hosts sampled/marked (integer) |
| AJ | NLocations | Number of individual location events (integer; for example, in a 1-year study of 10 animals each observed every week, this would be 520), in which the individual is the sampling unit (so this could refer to number of farms if the highest resolution of reported sampling is as the farm level). This field includes total number of radiotelemetry fixes, or total number of animal handling events. This will be approximately equal to NStepsInTimeSeries * NHostIndividuals if all animals are sampled at each timestep (and births/deaths are negligible). |
| AK | NDiseaseTests | Number of serological/PCR tests/animal handling events (integer). This will be equal to NLocations if all location events correspond to health sampling events. |
| AL | NStepsInTimeseries | Number of steps in average timeseries included in paper (if multiple locations and sites, NSites * NStepsInTimeseries * NHostIndivids should be approximately equal to NLocations). |
| AM | NSites | Number of separate physical regions within which sampling units occur (e.g., number farms or field sites surveyed if sampling unit is the individual, number regions/counties/etc. if sampling unit is the farm). |
| AN | CertainDataUsed | Is the amount of data used clearly detailed in the paper? (NA = not applicable / 0 = No / 1 = Yes / 2 = ambiguous). |
| AO | CovariatesIncluded | Does the model include non-demographic predictors e.g., habitat, climate, etc.? (1 = yes / 0 = no) |
| AP | VectorPopDynamics | Does the paper focus entirely on vector ecology? (1 = yes / 0 = no) |
| AQ | Evolutionary | Does the model pursue evolutionary questions? (1 = yes / 0 = no) |
| AR | Climate | Does the model pursue questions related to climate change? (1 = yes 0 = no) |
| AS | SoftwareUsed | What type of software was used? (Free text; e.g., R, Matlab, Mathematical, Maple, Python, C++, STATA, SAS, etc.) |
| AT | CustomProgramming** | Does this analysis contain custom programming (NA = not applicable / 0 = No / 1 = yes / 2 = unknown) |
| AU | ActualReader | Last name of actual reader of this paper (from among authors of this manuscript) |
| AV | NeedsSecondReader | Does paper need a second reader? ((0 = No / 1 = yes; say yes if many fields are unclear or paper is far outside your domain of expertise and you’re not confident in your responses) |

*Choices for model structure:

- - birth-death process model
  - generalized linear
  - generalized linear mixed effects
  - spatial process model (e.g., kriging a surface/ disease incidence mapping)
  - simple disease compartmental (e.g., SI, SIS, SIR, SEIR, SIRS, SEIRS but no additional heterogeneity) - population
  - disease compartmental - population - with age/sex structure
  - disease compartmental - population - with heterogeneous contact structure -- network (e.g., gravity models, cellular automata)
  - disease compartmental - population - with heterogeneous contact structure -- spatial (e.g., metapopulation models)
  - disease compartmental - population - with heterogeneous compartments (e.g., multiple ways to be “Infected” or “susceptible”)
  - disease compartmental - population - with seasonal forcing
  - disease compartmental - individual - with age/sex structure
  - disease compartmental - individual - with heterogeneous contact structure -- network (e.g., gravity models, cellular automata)
  - disease compartmental - individual - with heterogeneous contact structure -- spatial (e.g., metapopulation models)
  - disease compartmental - individual - with heterogeneous compartments (e.g., multiple ways to be “Infected” or “susceptible”)
  - disease compartmental - individual - l with seasonal forcing
  - Simple disease compartmental -- individual
  - Evolutionary -- game theory
  - Evolutionary -- phylogenetic / phylogeographic
  - Evolutionary -- strain competition
  - Evolutionary -- host/pathogen interactions
  - Evolution -- correlated transmission and virulence
  - MCMC Simulation
  - Risk assessment
  - Catalytic prevalence model
