# Supplementary material for: “One Health” or Three? Publication Silos Among the One Health Disciplines
Source: PLoS Biol. 2016 Apr 21;14(4):e1002448. doi: 10.1371/journal.pbio.1002448 (PMC4839662; doi:10.1371/journal.pbio.1002448)
Supplement: S1 Text — Detailed descriptions of data extraction protocols and analyses, as well as supporting results, including tables and figures that complement the main text. (DOCX) [file pbio.1002448.s023.docx]

**“One Health” or Three? Publication Silos Among the One Health Disciplines**

**S1 Text**

**Contents**

1. Search methods ----------------------------------------------------------------------------------------------- 2

1. Network construction and descriptions ------------------------------------------------------------------- 2
2. Supplemental methods text -------------------------------------------------------------------------------- 3
3. Specific effect sizes from Fig 4 --------------------------------------------------------------------------- 6

5. List of journals in each community ----------------------------------------------------------------------- 7

6. Ten most-cited papers in each community -------------------------------------------------------------- 10

7. References ---------------------------------------------------------------------------------------------------- 12

1. **Search methods**

Search terms (S1 Text Table S1) were initially applied to the 25 best-ranked journals in each designated category (S1 Text Table S2) in the Scientific Journal Rankings from 2013 as determined by SciImago (<http://www.scimagojr.com/journalrank.php?country=US>). The initial search (run Nov. 4, 2014) returned 1651 papers, which we used to build a database consisting of citations from those 1651 papers. We augmented our journal list to include any journal excluded from the original journal list, but referenced more than 250 times by papers selected under the initial search (added journals listed in S1 Text Table S3). This augmentation allowed us to incorporate lower-impact journals that still publish extensively on infectious disease transmission.

We reran the original search on the augmented journal list to achieve our final paper list on Nov. 4, 2014. This search returned 2258 papers. A single reader read abstracts of all returned papers and excluded papers with no direct or indirect reference to disease transmission in the title or abstract. A second reader read abstracts from all papers excluded by the first reader to confirm exclusion. Following abstract review, the paper list was reduced to 1605 papers, which formed our paper bank and the basis for the descriptions below.


**2. Network construction and descriptions**

Journals

Journals identified by journal name

Nodes = journals that published papers included in search;

Edges link journals whose papers cite one another (asymmetric edges weighted by frequency of citations from Journal A of papers in Journal B)

Papers

Papers identified by DOI (when available), or title and year published (when DOI not available)

Nodes = all papers included in reduced paper bank;

Edges link papers that cite each other in search (directed, binary edges – in only instance did two papers reciprocally cite each other).

Authors

Authors identified by last name + first initial

Nodes = all authors who wrote papers included in the reduced paper bank

Edges link authors who coauthored a manuscript (symmetric and weighted by frequency of coauthorship)

1. **Supplemental Methods Text**

The search was limited to the 25 highest-ranked journals identified by SciMago (1) in nine different subdisciplines related to infectious diseases (listed in Table S2). We extracted all references from papers captured in a first-round search of Web of Science, and expanded the search to include any additional journals that were cited more than 250 times in the preliminary search, but that were not ranked in the top 25 of their particular subdisciplines (see added journals in Table S3). We reran the search on this expanded journal list in Web of Science. A primary reader (KM) read abstracts from all papers in the initial complete paper list. Papers that did not directly reference disease transmission in the abstract or title were eliminated. A second reader (JGW) reviewed all eliminated abstracts to confirm that elimination was appropriate. This list of papers (n = 1628) constituted our paper bank.

We extracted Digital Object Identifiers (DOIs), lead author surname and first initial, and year of publication for each reference cited by each paper in our paper bank. Then, we searched the paper bank for papers that matched cited DOIs (when available) or lead author/year combinations. When matches were discovered, we assigned a directed edge linking the citing paper to the paper cited. Next, we built a coauthorship network linking all authors (nodes) in the paper bank along edges defined by coauthorship. A third network of journal communities described citations (edges) between the journals (nodes) included in the paper bank.

Network topology

The journal and paper networks were directed, so relationships between nodes were not usually symmetric (for example, Paper A can cite Paper B without B citing A); the author network had symmetric edges. The author and journal networks both had weighted edges reflecting the frequency of coauthorship or citations, respectively, but edges in the paper network were binary.

We first identified (weakly) connected components, sets of nodes that can be accessed from one another, in each network. We also characterized each network in terms of its average degree (the mean number of edges attached to each node), diameter (the length of the longest non-looping path between connected nodes), and average path length (the mean number of steps separating any pair of nodes in the network). Average degree describes how many connections each node has on average. Diameter reflects how compactly a network is structured. Low values mean that all nodes are closely tied to one another. Average path length describes the average number of steps separating a pair of nodes. All network analyses were conducted using the igraph package (2) in R (3).

Author affiliations and research discipline diversity

For authors that contributed multiple papers to the paper bank or had multiple affiliations, we relied on the oldest affiliation or first-listed, which we presumed best-reflected the author’s primary training. Lead author affiliations and corresponding discipline assignments are included in Supplementary Dataset 1. We also used paper metadata to assign disciplines to each co-author. Specifically, we conducted a text search of author affiliations for the following strings: “stat”, “math”, “eco”, “evo”, “biol”, “vet”, “med”, and “epi”. We reviewed all lead-author affiliations, and added the following domain categories: animal science/animal health; health informatics; governmental domains; other academic research disciplines (anthropology, chemistry, economics, psychology, sociology); and pharmacy. Authors with multiple domain affiliations were counted under each identifiable discipline, and papers whose lead author affiliation could not be clearly assigned to a particular discipline were excluded from author-affiliation analyses (true for 58 of the 1628 papers, or 3.5%).

Diversity models

We measured author disciplinary diversity within particular papers, and lead author disciplinary diversity within journal communities using Shannon’s diversity index, $H',$ as implemented through the diversity function in the R package vegan (4). Shannon’s diversity was used to measure author diversity in two ways. First, we measured author diversity on particular papers through time, and second, we measured lead author diversity within each journal community through time. Shannon’s diversity is calculated as follows. If a group has *I* distinct categories, each represented at proportion $p_{i}$, then $H^{'}=\sum_{i=1}^{I} p_{i}log\left( p_{i} \right).$ We distinguished between lead-author diversity within a journal community, ${H'}_{L}$, and diversity among co-authors on each paper, ${H'}_{C}.$ We examined patterns between each measure of author diversity and number of authors on the paper (for ${H'}_{C}$) or papers in the community (for ${H'}_{L}$) to determine whether trends were driven by increasing numbers of authors or numbers of papers through time (Fig. S5).

We used AIC (5) to compare models that allowed different year-diversity relationships and different baseline diversities for each community to models that assumed common baselines and year-diversity relationships. The second model characterized how citations between journal communities and citations within journal communities vary between communities and over time. In both models, the primary predictor, year of publication, was incorporated through smoothing splines. In each case, the model included an additive shift due to membership of individual$i$in the$j^{th}$community ($\alpha_{j\left[ i \right]}$), a spline function describing changes over years ($s_{1}$), and a spline term capturing interaction between year and community ($s_{2}$), where community is denoted by indicator variables ${Comm}_{1},{Comm}_{2}$, and ${Comm}_{3}.$ In full, the author diversity of the $i^{th}$ observation (${{H'}_{L}}_{i}$ and ${{H'}_{C}}_{i}$) within journal community $j$ was modeled as

${H'}_{i}=\alpha_{j\left[ i \right]}+s_{1}\left( {Year}_{i} \right)+s_{2}\left( {Year}_{i} \right)\times{Comm}_{j\left[ i \right]}+\varepsilon_{i}$,

where $\varepsilon_{i}\sim N\left( 0,\sigma^{2} \right)$. We compared models that fit separate splines for each journal community to a model that only allowed community intercepts to vary. Models were fit in the mgcv package (6) in R.

Citation rate model

We identified factors leading to high citation rates by modeling the total number of times each of the 1628 papers in the full paper bank was cited (denoted$Y_{i}$, such that $Y_{i}\sim Poisson\left( \lambda_{i} \right)$), as a function of that paper's within-paper authorship diversity (${H'}_{C}$), citation diversity ($R,$the ratio of between-community citations to total citations), and publication year ($Year$). The model allowed for community-specific intercepts ($\alpha_{j\left[ i \right]}$), slopes associated with author diversity ($\delta_{j\left[ i \right]}$), and slopes associated with citation diversity ($\phi_{j\left[ i \right]}$). The model had a log-link and an offset term for years since publication.

$$\lambda_{i}=exp\left[ \alpha_{j\left[ i \right]}+\beta{Year}_{i}+\delta_{j\left[ i \right]}{{H'}_{C}}_{i}+\phi_{j\left[ i \right]}R_{i}+\varepsilon_{j} \right]$$

Models were fitted using the glm function from R’s stats package.

Community identification and stratified sampling

Strata were defined by journal community, annual paper citation rate (lowest 20% within community, 40th to 60th percentile within community, highest 20% within community), and year of publication in detail. We binned year of publication into three four-year blocks (1998-2002; 2003-2007; 2008-2012).

Years were chosen to capture long-term trends in collaboration, while still incorporating/including a sufficient number of papers within each journal community. See Table S4 for the number of papers in each stratum. A team of 10 readers read 20 articles each within these nine strata and extracted information on 25 different variables (listed in Table S5). When extracted information was ambiguous, papers were read and independently scored by a second reader from the team. To compare inter-reader variation, a pre-selected subsample of 35 papers was read by two readers. We estimated binomial confidence intervals and used them to compare features of the literature from the three journal communities through time.

Finally, we identified highly cross-disciplinary journals in dynamic modeling of infectious disease by comparing the composition of lead author affiliations across all papers in our paper bank to the composition of lead author affiliations within paper bank papers from each particular journal. We calculated the deviance of lead author affiliations within each journal from the overall distribution of lead authors to identify journals whose author affiliation frequencies reflected frequencies over the entire paper bank.

1. **Specific effect sizes from Fig 4**

Effect of increasing author diversity from 0 to 1 in the ecology community was (exp(β) = 1.64; SE = 0.27). The same effect in the human-focused epidemiology community was (exp(β) = 1.08; SE = 0.05); and in the veterinary community it was (exp(β) = 1.26; SE = 0.08). All three effects were highly significant (p < 0.001 in all cases). The effect of increasing citations from entirely internal to entirely external in the ecology community was (exp(β) = 1.03; SE = 0.03; p = 0.371); in the veterinary community it was (exp(β) = 0.654; SE = 0.10; p-value of interaction term < 0.001); in Group 3, this effect was (exp(β) = 0.95; SE = 0.07; p-value of interaction term = 0.072).


**5. List of journals in each community**

Journals in Ecology Community

| **Journal Name** | **Number of papers contributed to bank** |
| --- | --- |
| American Naturalist | 30 |
| Annual Review of Entomology | 1 |
| Applied and Environmental Microbiology | 6 |
| Biological Conservation | 3 |
| Biological Reviews | 1 |
| Bioscience | 2 |
| BMC Biology | 2 |
| Conservation Biology | 5 |
| Current Opinion in Microbiology | 1 |
| Ecological Applications | 13 |
| Ecological Modelling | 35 |
| Ecology | 24 |
| Ecology Letters | 24 |
| Evolution | 14 |
| Evolutionary Applications | 3 |
| Frontiers in Zoology | 1 |
| Integrative and Comparative Biology | 3 |
| Journal of Animal Ecology | 19 |
| Journal of Applied Ecology | 33 |
| Journal of Ecology | 4 |
| Journal of Mammalogy | 1 |
| Journal of Wildlife Diseases | 9 |
| Journal of Wildlife Management | 7 |
| Landscape Ecology | 4 |
| Molecular Ecology | 1 |
| Parasitology | 14 |
| Philosophical Transactions of the Royal Society B - Biological Sciences | 10 |
| Phytopathology | 36 |
| PLoS Biology | 2 |
| Zoonoses and Public Health | 2 |

Journals in Human-Focused Epidemiology Community

| **Journal Name** | **Number of papers contributed to bank** |
| --- | --- |
| American Journal of Epidemiology | 27 |
| American Journal of Preventive Medicine | 1 |
| American Journal of Tropical Medicine and Hygiene | 25 |
| Behavioral Ecology and Sociobiology | 2 |
| Biometrics | 10 |
| Chinese Science Bulletin | 1 |
| Clinical Microbiology and Infection | 4 |
| Current Opinion in Infectious Diseases | 1 |
| Ecological Complexity | 13 |
| Ecological Informatics | 1 |
| Ecological Monographs | 3 |
| Epidemics | 26 |
| Epidemiology | 11 |
| Epidemiology and Infection | 46 |
| Genetics | 5 |
| Global Change Biology | 2 |
| Infection Control and Hospital Epidemiology | 6 |
| International Journal of Epidemiology | 6 |
| Journal of Clinical Microbiology | 1 |
| Journal of Infectious Diseases | 4 |
| Journal of Mathematical Biology | 41 |
| Journal of Theoretical Biology | 102 |
| Journal of the Royal Society Interface | 79 |
| Mathematical Biosciences | 84 |
| Molecular Biology and Evolution | 6 |
| Nature | 12 |
| PLoS Medicine | 9 |
| PLoS Neglected Tropical Diseases | 46 |
| PLoS One | 169 |
| Proceedings of the National Academy of Sciences USA (PNAS) | 52 |
| Proceedings of the Royal Society B - Biological Sciences | 68 |
| Science | 6 |
| Scientific Reports | 8 |
| Statistical Methods in Medical Research | 2 |
| Statistics in Medicine | 24 |
| Theoretical Ecology | 6 |
| Theoretical Population Biology | 35 |
| Trends in Microbiology | 2 |
| Vaccine | 53 |

Journals in Veterinary Medical Community

| **Journal Name** | **Number of papers contributed to bank** |
| --- | --- |
| American Journal of Veterinary Research | 12 |
| Journal of Dairy Science | 4 |
| Preventive Veterinary Medicine | 138 |
| Transboundary and Emerging Diseases | 10 |
| Veterinary Microbiology | 13 |
| Veterinary Record | 2 |
| Veterinary Research | 19 |

**6. Ten most-cited papers in each community**

Ecology

1. Altizer S, Dobson A, Hosseini P, Hudson P, Pascual M, Rohani P (2006) Seasonality and the dynamics of infectious disease. *Ecology Letters* 9: 467-484.
2. Van Baalen M, Sabelis MW (1995) The dynamics of multiple infection and the evolution of virulence. *American Naturalist* 146(6): 881-910.
3. Gage KL, Kosoy MY (2005) Natural history of plague: Perspectives from more than a century of research. *Annual Review of Entomology* 50: 505-528.
4. Roy BA. Kirchner JW (2000) Evolutionary dynamics of pathogen resistance and tolerance. *Evolution* 54(1): 51-63.
5. deCastro F, Bolker B (2005) Mechanisms of disease-induced extinctions. *Ecology Letters* 8(1): 117-126.
6. Chan MS, Medley GF, Jamison D, Bundy DAP (1994) The evaluation of potential global morbidity attributable to intestinal nematode infections. *Parasitology* 109: 373-387.
7. Miller MW, Williams ES, McCarty CW, Spraker TR, Kreeger TJ, Larsen CT, Thorne ET (2000) Epizootiology of chronic wasting disease in free-ranging cervid*s* in Colorado and Wyoming. *Journal of Wildlife Diseases* 36(4): 676-690.
8. Smith DL, McKenzie FE, Snow RW, Hay SI (2007) Revisiting the basic reproductive number for malaria and its implications for malaria control. *PLoS Biology* 5(4): 531-542.
9. Woods A, Coates KD, Hamann A (2005) Is an unprecedented dothistroma needle blight epidemic related to climate change? *Bioscience* 55(9): 761-769.
10. Ebert D, Lisitch M, Mangin KL (2000) The effect of parasites on host population density and extinction: Experimental epidemiology in Daphnia and six microparasites. *American Naturalist* 156(5): 459-477.

Human-Focused Epidemiology

1. Longini IM, Nizam A, Xu SF, Ungchusak K, Hanshaoworakul W, Cummings DAT, Halloran ME (2005) Containing pandemic influenza at the source. *Science* 309(5737): 1083-1087.
2. Germann TC, Kadau K, Longini IM, Macken CA (2006) Mitigation strategies for pandemic influenza in the United States. *PNAS* 103(15): 5935-5940.
3. Lloyd-Smith JO, Schreiber SJ, Kopp PE, Getz WM (2005) Superspreading and the effect of individual variation on disease emergence. *Nature* 438(7066): 355-359.
4. Keeling MJ, Woolhouse MEJ, Shaw DJ, Matthews L, Chase-Topping M, Haydon DT, Cornell SJ, Kappey J, Wilesmith J, Grenfell BT (2001) Dynamics of the 2001 UK foot and mouth epidemic: Stochastic dispersal in a heterogeneous landscape. *Science* 294(5543): 813-817.
5. Longini IM, Halloran ME, Nizam A, Yang Y (2004) Containing pandemic influenza with antiviral agents. *American Journal of Epidemiology* 159(7): 623-633*.*
6. Mills CE, Robins JM, Lipsitch M (2004) Transmissibility of 1918 pandemic influenza.  *Nature* 432(7019): 904-906*.*
7. Hufnagel L, Brockmann D, Geisel T (2004) Forecast and control of epidemics in a globalized world. *PNAS* 101(42): 15124-15129*.*
8. Ferguson NM, Donnelly CA, Anderson RM (2001) The foot-and-mouth epidemic in Great Britain: Patterns of spread and impact of interventions. *Science* 292(5519):1155-1160*.*
9. Keeling MJ (1999) The effects of local spatial structure on epidemiological invasions. *Proc R Soc B* 266(1421): 859-867.
10. Colizza V, Barrat A, Barthelemy M, Vespignani (2006) The role of the airline transportation network in the prediction and predictability of global epidemics. *PNAS* 103(7): 2015-2020*.*

Veterinary

1. Morris RS, Wilesmith JW, Stern MW, Sanson RL, Stevenson MA (2001) Predictive spatial modelling of alternative control strategies for the foot-and-mouth disease epidemic in Great Britain, 2001. *Veterinary Record* 149(5): 137.
2. Groenendaal H, Nielen M, Jalvingh AW, Horst SH, Galligan DT, Hesselink JW (2002) A simulation of Johne’s disease control. *Preventive Veterinary Medicine* 54(3): 225-245.
3. Jalvingh AW, Nielen M, Maurice H, Stegeman AJ, Elbers ARW, Dijkhuizen AA (1999) Spatial and stochastic simulation to evaluate the impact of events and control measures on the 1997-1998 classical swine fever epidemic in the Netherlands. I. Description of simulation model. *Preventive Veterinary Medicine* 42(3-4): 297-317*.*
4. Garner MG, Lack MB (1995) An evaluation of alternate control strategies for foot-and-mouth disease in Australia - A regional approach. *Preventive Veterinary Medicine* 23(1-2): 9-32*.*
5. Bates TW, Thurmond MC, Carpenter TE (2003) Description of an epidemic simulation model for use in evaluating strategies to control an outbreak of food-and-mouth disease. *American Journal of Veterinary Research* 64(2): 195-204*.*
6. Alban L, Stark KDC (2005) Where should the effort be put to reduce the Salmonella prevalence in the slaughtered swine carcass effectively? *Preventive Veterinary Medicine* 68(1): 63-79*.*
7. Nielen M, Jalvingh AW, Meuwissen MPM, Horst SH, Dijkhuizen AA (1999) Spatial and stochastic simulation to evaluate the impact of events and control measures on the 1997-1998 classical swine fever epidemic in the Netherlands. II. Comparison of control strategies. *Preventive Veterinary Medicine* 42(3-4): 297-317.
8. Barlow ND, Kean JM, Hickling G, Livingstone PG, Robson AB (1997) A simulation model for the spread of bovine tuberculosis within New Zealand cattle herds. *Preventive Veterinary Medicine* 32(1-2): 57-75.
9. Doran RJ, Laffan SW (2005) Simulating the spatial dynamics of foot and mouth disease outbreaks in feral pigs and livestock in Queensland, Australia, using a susceptible-infected-recovered dellular automata model. *Preventive Veterinary Medicine* 70(1-2): 133-152.
10. Stegeman A, Elbers ARW, Smak J, de Jong MCM (1999) Quantification of the transmission of classical swine fever virus between herds during the 1997-1998 epidemic in The Netherlands. *Preventive Veterinary Medicine* 42(3-4): 219-234.


**7. Referencess**

1.SCImago. (2007). SJR — SCImago Journal & Country Rank. Retrieved November 14, 2014, from [http://www.scimagojr.com](http://www.scimagojr.com/)

2. Csardi G, Nepusz T (2006) The igraph software package for complex network research. *InterJournal, Complex Systems* 1695. [http://igraph.org](http://igraph.org/)

3. R Core Team (2014) R: A language and environment for statistical computing. R Foundation for Statistical Computing, Vienna, Austria. [http://www-R-project.org/](http://www-r-project.org/).

4. Oksanen, J et al. (2015) vegan: Community Ecology Package. R package version 2.3-0. [http://CRAN.R-project.org/package=vegan](http://cran.r-project.org/package=vegan)

5. Burnham K, Anderson D (2002) *Model Selection and Multi-model Inference*. Springer-Verlag, New York. 10.1007/b97636.

6. Wood SN (2011) Fast stable restricted maximum likelihood and marginal likelihood estimation of semiparametric generalized linear models. *J Roy Stat Soc B* 73(1): 3-36.
